# NLRP3 receptor contributes to protection against experimental antigen-mediated cholangitis

**DOI:** 10.1042/BSR20200689

**Published:** 2020-08-07

**Authors:** Marisol Ibet González, Danielle Vannan, Bertus Eksteen, José Luis Reyes

**Affiliations:** 1 Laboratorio de Inmunología Experimental y Regulación de la Inflamación Hepato-Intestinal, UBIMED, FES Iztacala, UNAM. Tlalnepantla de Baz, Estado de México, C.P. 54090, México; 2 Boston Scientific 300 Boston Scientific Way Marlborough, MA, U.S.A.; 3 Aspen Woods Clinic, 8561 8A Ave SW Calgary AB T3H 0V5, Canada

**Keywords:** Cholangitis, Liver inflammation, NLRP3

## Abstract

Inflammatory diseases of the bile ducts like primary sclerosing colangitis (PSC) are characterized by a robust cellular response targeting the biliary epithelium leading to chronic inflammation and fibrosis. Driving fibro-inflammatory diseases, NOD-like receptors such as NLRP3 have been identified as a central component to immune-mediated pathology. However, to date the role of NLRP3 in biliary diseases has been poorly explored. Here, we addressed the role of NLRP3 in the OVAbil mouse model of antigen-mediated cholangitis. As obesity continues to spread worldwide, we also evaluated the NLRP3 response in experimental cholangitis after high-fat diet exposure**.** We compared the extent of histopathological liver damage between OVAbil and OVAbilxNLRP3^−/−^ mice after either a standard chow or a high-fat diet. Infiltrating immune cells were characterized by flow cytometry and levels of cytokines, chemokines and liver enzymes in blood samples were analyzed at the end of the experiment. We observed a more severe histopathological phenotype of cholangitis in absence of NLRP3, characterized by loss of bile ducts and larger inflammatory foci and higher levels of IL- 6 and CXCL10 as compared with NLRP3 sufficient mice. This phenotype was further exaggerated in the context of obesity, where cholangitis induced in NLRP3-deficient obese mice resulted in further exacerbated histopathology and increased levels of IL-13 and TNFα, suggesting a diet-specific profile. The absence of NLRP3 caused a supressed IL-17 response. In summary, our data suggest that activation of NLRP3 attenuates this antigen-mediated OVAbil model of cholangitis.

## Introduction

Biliary diseases such as primary sclerosing cholangitis (PSC) are perpetuated by a chronic inflammatory response targeting the biliary epithelial cells (BECs), which if not controlled, may lead to a strong fibrotic response and liver failure [1]. Therefore, identifying and controlling inflammation to prevent evolution of severe irreversible damage, including neoplasia, has become a main target in biliary disease research. One of the central components of the early inflammatory response is the inflammasome. NOD-like receptor pyrin domain-containing protein 3 (NLRP3) is a cytoplasmic sensor, with the ability of assembly multiproteic platforms known as inflammasomes that serve as docking sites for caspase 1 which ultimately cleaves the cytokines IL-1β and IL-18 [2]. Emerging evidence shows that NLRP3 inflammasome may play disease-specific roles in the gastrointestinal (GI) tract; however, conclusions from the literature remain controversial. For instance, numerous studies have shown that attenuation of DSS-colitis, a closely related disease to PSC, might be achieved by repressing the NLRP3 inflammasome [3–7]. However, contradicting results have been reported in early experiments using NLRP3-deficient mice [8–10] where the gut microbiota is thought to be largely responsible for the opposing effects [11]. In the context of biliary diseases, several reports suggest the involvement of NLRP3 as demonstrated in reactive cholangiocytes from PSC patients and cell lines expressing NLRP3 [12]. Moreover, bile acids such as deoxycholic acid (DCA) [13] and chenodeoxycholic acid (CDCA) [14] induce NLRP3 expression, this latter leading to cholestatic liver injury and fibrosis. Thus, existing data describing the role of NLRP3 in inflammatory diseases of the GI tract remain highly complex and controversial.

Here, we describe that NLRP3 is required to limit the immunopathogy observed in experimental antigen-specific biliary inflammatory disease. Mice lacking the NLRP3 inflammasome exhibited a more severe bile duct destruction, which was exacerbated when mice were exposed to an obesogenic diet.

## Materials and methods

### Mice and cholangitis induction

We modeled acute cholangitis by using a slightly modified protocol from the OVAbil model [15] as previously reported [16]. Animal experiments were conducted in the Snyder Institute for Chronic Diseases (University of Calgary) and the study protocol M11025 was approved by the University of Calgary Animal Care Committee and conformed to the *Guidelines for the Care and Use of Laboratory Animals.* Briefly, C57BL/6 male mice expressing fragment 139-285aa of the membrane-bound ovalbumin protein (OVAbil) were used as recipients animals and compared to age- and sex-matched mice lacking the NLRP3 sensor (OVAbilxNLRP3^−/−^). Both groups were fed either a standard chow (SC) or a high-fat diet (HFD) for 12 weeks as previously described [16]. Both SC and HFD-fed mice received 1×10^7^ splenic lymphocytes obtained from OVA-transgenic mice I and II (OTI and OTII), via intraperitoneal (ip.) injections.

### Histology

Ten days post-transfer of OTI and OTII cells (cholangitis induction) mice were humanly killed under deep inhaled anesthesia (isoflurane) followed by cervical dislocation and liver samples collected. Tissue was paraffin-embeded and sectioned (5 µM thick) before proceding to stain with H&E for histopathological analysis. Under the light microscope, 5 portal tracts chosen at random were scored for bile duct architecture as follows: 0; bile duct well-preserved, 1; disarranged bile duct 2; infiltrating cells displacing cholangiocytes, 3; bile duct absent. For inflammation assesssment 0; no infiltrating cells, 1; moderate infiltrating cells, 2; massive inflammatory cells only in portal tract 3; massive inflammatory cells including liver parenchyma.

### Flow cytometry

At sacrifice, livers from experimental groups were retrieved and homogenized in FBS-containing PBS using a gentleMACS tissue dissociator (Miltenyi Biotec Inc, Germany). Samples were layered onto 33/77% percoll gradients (Percoll, GE Healthcare Bio-Sciences AB, Uppsala Sweden) and centrifuged for 20 min at 200 ***g***, at room temperature to achieve cell separation. Cell suspensions recovered from the gradients were adjusted (1 × 10^6^/ml) and stained in flow cytometry buffer (5%FBS, 5mM EDTA and 0.1% sodium azide). Staining was conducted using the following fluorochrome-conjugated antibodies: PO-CD11b, PECy7-F4/80, APC-Cy7-Ly6C and PE-Ly6G. Data were acquired on a flow cytometer Aria II (BD Biosciences) and analyzed with Kaluza software (Beckman Coulter, U.S.A.).

### Serum tests

Whole blood was collected via cardiac puncture from mice under deep anesthesia at sacrifice (Isoflurane, Halocarbon products). Blood samples were centrifuged and sera from experimental groups were tested for alanine aminotransferase (ALT) levels by Calgary Laboratory Services (Calgary, AB, Canada) and soluble immune mediators with the mouse cytokine 31-plex Discovery Assay (Eve Technologies, Calgary, AB, Canada) as reported [16]. Cytokine and chemokine concentrations are shown as pg/ml.

### Data presentation

All results are presented as mean ± standard error of the mean (SEM). Data were analyzed in Graph pad prism 6.0 software by one way ANOVA followed by multiple comparisons where a *P*<0.05 was accepted as significant.

## Results

**Figure 1:**
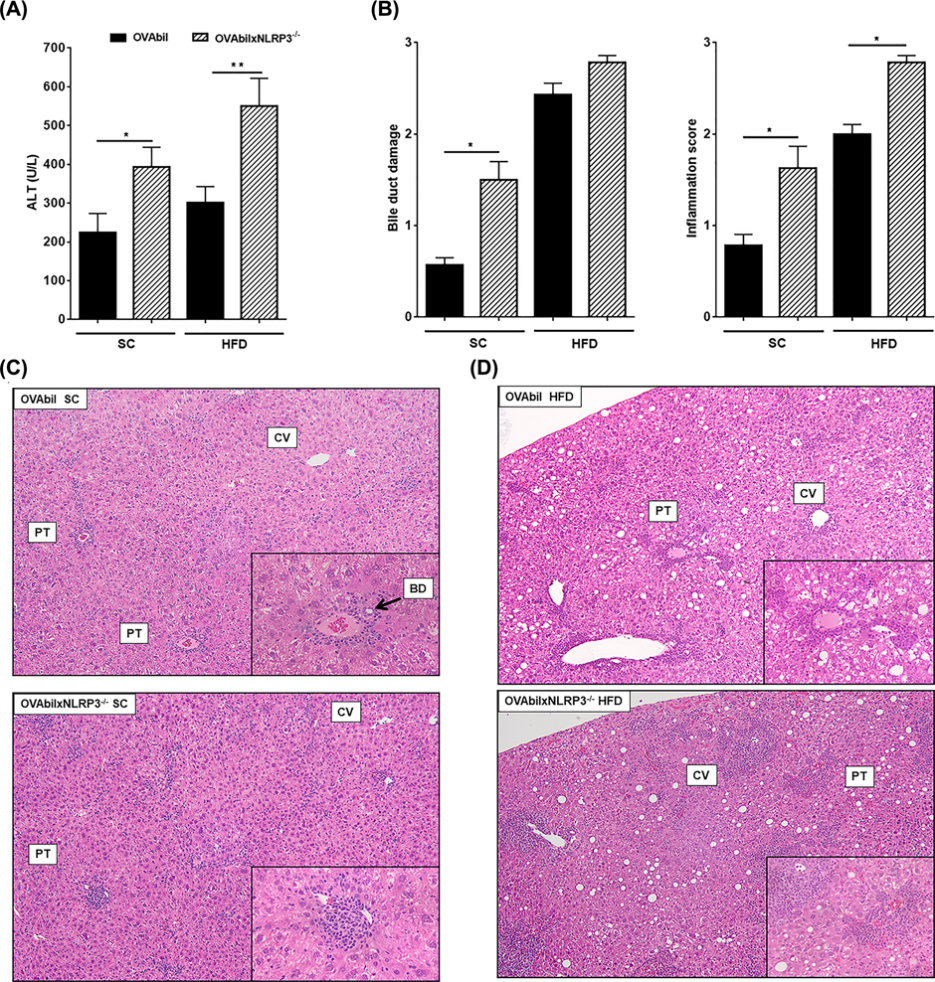
The presence of NLRP3 prevents bile duct loss Eight-week old mice from experimental groups (OVAbil vs OVAbilxNLRP3^−/−^) fed a standard chow were given 1 × 10^7^ from each OVA-specific CD4 and CD8 T cells donors. Mice fed a high fat diet for 12 weeks receiving the same transfer protocol were also compared. At the peak of disease (10 days post-transfer), serum samples were collected in order to determine ALT levels (**A**). Liver samples were stained and histological damage assessed by means of bile duct loss and inflammatory cell infiltration (**B**), representative images of SC-fed mice (**C**) and HFD-fed mice (**D**) are shown. Data shown are representative from two experiments (*n*=8–11) where *P<0.05 and **P<0.01. BD; bile duct, CV; central vein, PV; portal vein.

The OVAbil model of immune-mediated cholangitis has a well-characterized immune response pattern that ultimately results in bile duct destruction at disease peak 10 days post-transfer of OVA-specific T cells [15]. Herein, we analyzed the extent of injury in the portal tracts as well as changes in liver myeloid cell distribution and circulating cytokine profiles at the peak of disease. Following transfer of OVA-transgenic T cells, we observed a significant increase in levels of serum ALT in OVAbilxNLRP3^−/−^ mice as compared to OVAbil (393 ± 51 vs 224 ± 49, respectively, see Figure 1A). Histopathological analysis showed a moderate degree of infiltrating immune cells in SC OVAbil mice on day 10 post-transfer of OVA-Tcells with visible bile ducts in the portal tracts (Figure 1B,C). In contrast, OVAbilxNLRP3^−/−^ had a wider inflammatory foci at the portal tracts and a loss of identifiable bile ducts (Figure 1B,C). Next, we decided to test the hypothesis of that the absence NLRP3 inflammasome may be selectively affecting our OVAbil model since it has been previously reported that NLRP3 is involved in the development of obesity and liver steatosis [17]. In line with our previous findings [16], OVAbil mice fed an obesogenic diet exhibited heightened infiltrating immune cells targeting cholangiocytes resulting in absent bile ducts (Figure 1D). Interestingly, OVAbilxNLRP3^−/−^ mice developed comparable steatosis as OVAbil mice did, however, a larger area of periportal and more frequent parenchymal inflammatory foci were observed (Figure 1D).

**Figure 2:**
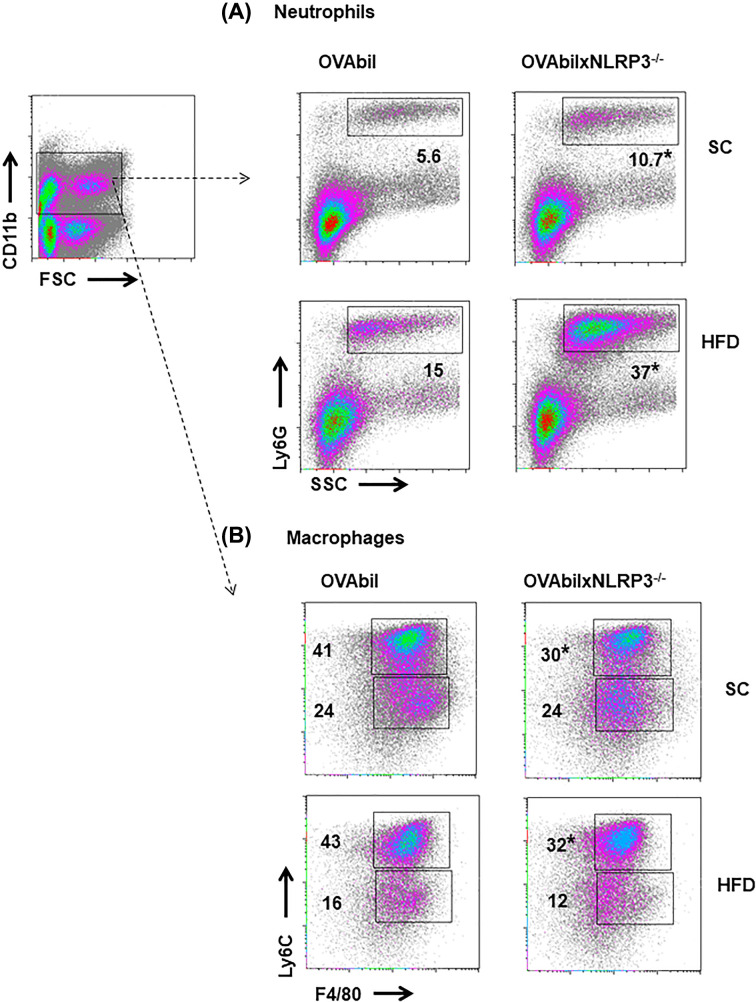
Massive infiltration of neutrophils correlates with severe biliary damage At peak bile duct injury induction excised liver samples were homogenized and total cells collected and adjusted from Percoll gradients. Cells from the myeloid gate (CD11b^+^) were further identified as Ly6G^+^SSC^+^ neutrophils seen in upper panel (**A**) and F480^+^Ly6C^hi^ infiltrating- and F480^+^Ly6C^lo^ resident macrophages (**B**). Data shown are representative from two experiments (*n*=5–7) where *P<0.05 as compared with OVAbil mice receiving the same pathogenic T cells.

We previously reported that the severity of our model of cholangitis is associated with neutrophil infiltration and decreased numbers of monocytes [16]. Here, we confirmed that an exaggerated histological injury, which ocurred in absence of NLRP3 in OVAbil mice, was accompanied with massive recruitment of neutrophils (Figure 2A). In line with this, obese OVAbilxNLRP3^−/−^ mice showed an even more dramatic increase in neutrophil numbers (Figure 2A, lower pannel). When distribution of Ly6C^hi^ and Ly6^lo^ macrophages was also determined within the myeloid population (CD11b^+^), we observed lower numbers of the Ly6C^hi^ macrophage sub-population in mice lacking NLRP3 regardless of the diet used (Figure 2B). Thus, OVAbil mice lacking NLRP3 had exacerbated numbers of neutrophils which correlated with an uncontrolled reponse targeting cholangiocytes.

**Figure 3:**
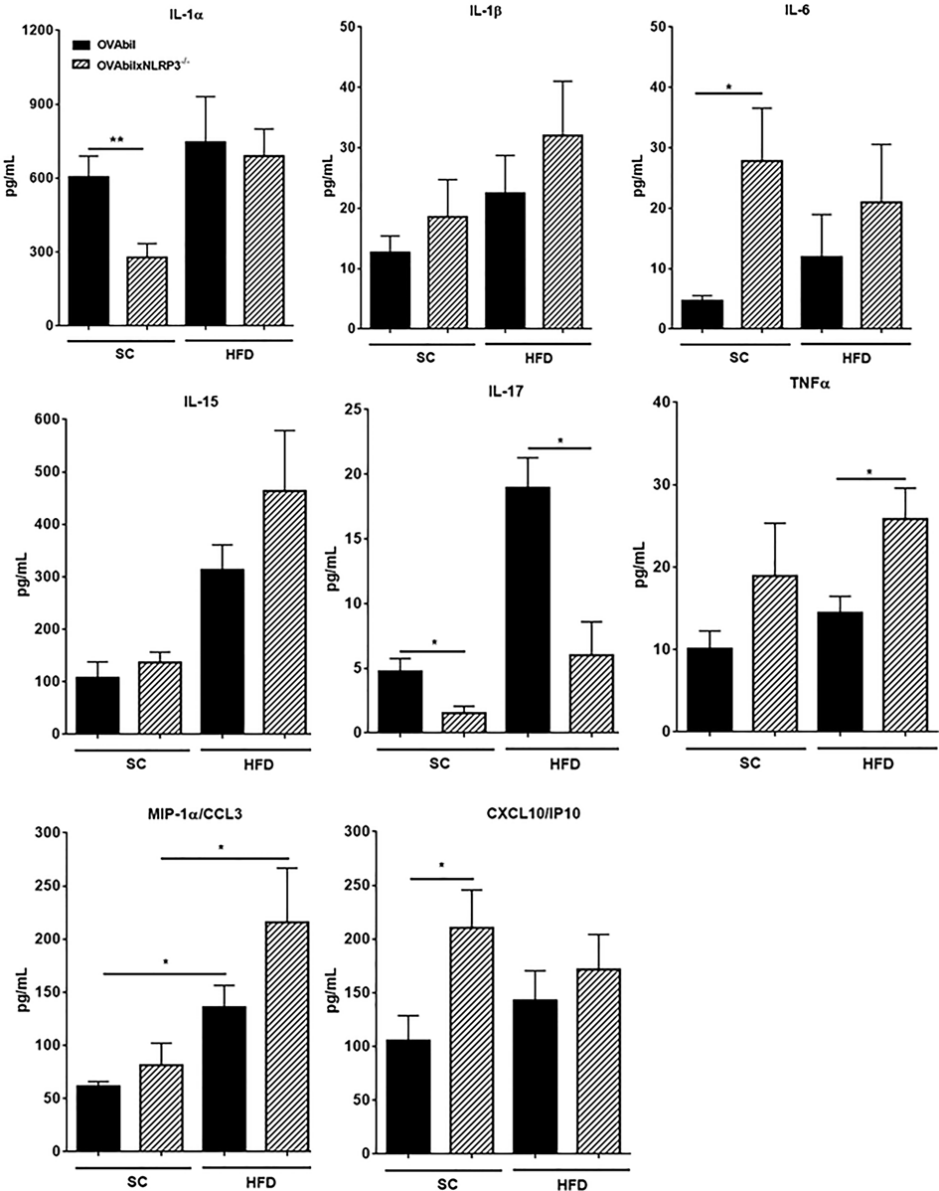
Diet-dependent profile of inflammatory mediators lead to biliary inflammation On the day of sacrifice whole blood was obtained by cardiac puncture centrifuged and sera tested for the above mentioned analytes. Data shown are mean ± SEM from two experiments (*n*=8–11), where *P<0.05 as compared with their diet-matched counterparts.

**Figure 4:**
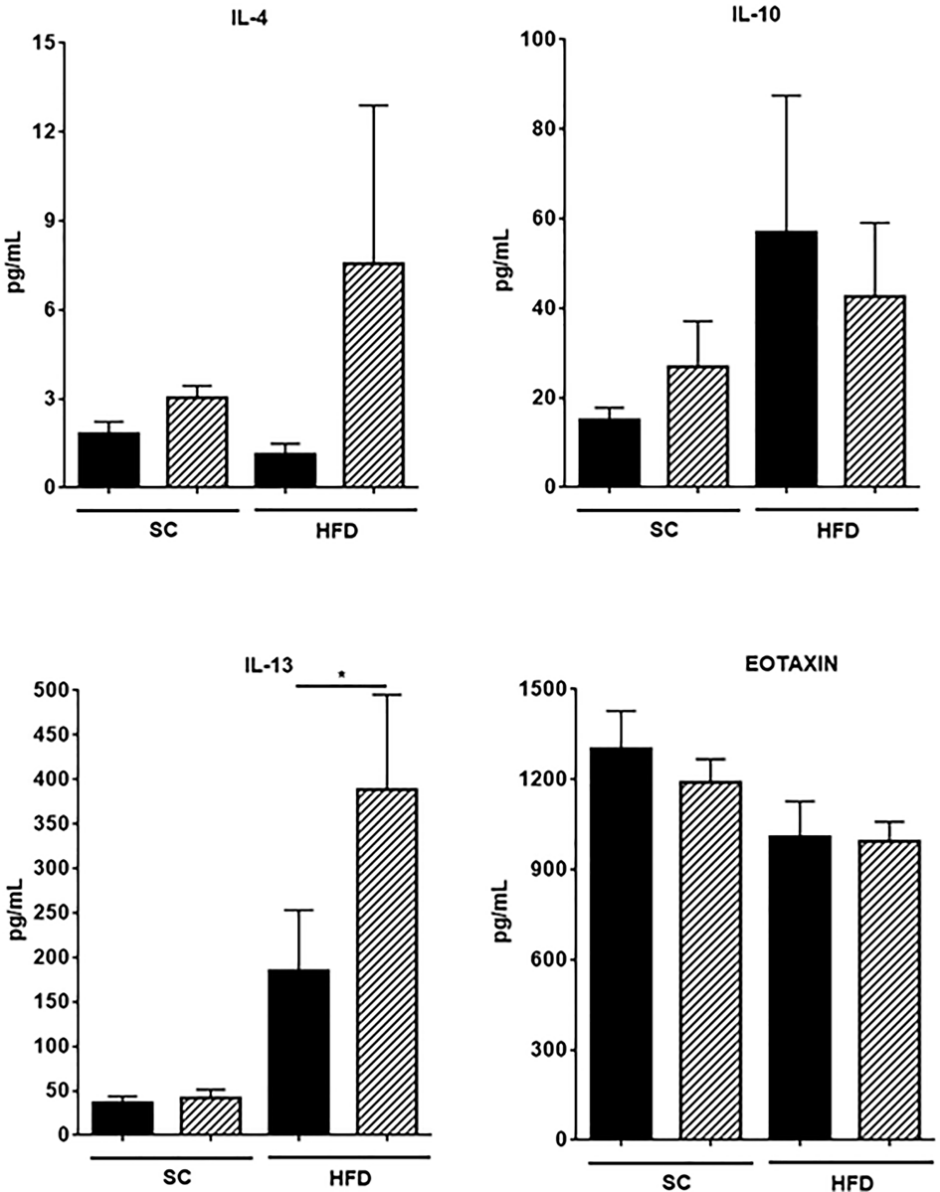
Type 2-associated mediators in serum samples Ten days post-transfer of pathogenic T cells blood samples were collected from experimental groups and assayed for cytokines and chemokines as indicated in material and methods. Levels of IL-4, IL-10 IL-13 and eotaxin are depicted. Data shown are mean ± SEM from two experiments (*n*=8–11), where *P<0.05.

To gain insight into changes in cytokines and chemokines as a consequence of NLRP3 deficiency in mice with experimental cholangitis, blood samples were tested for the indicated cytokines (Figure 3). Interestingly, we found higher levels of IL-1α as compared with IL-1β levels in our experimental groups. We noticed a significant reduction in IL-1α in blood samples from OVAbilxNLRP3^−/−^ mice as compared to those samples obtained from OVAbil mice but only under a regime of SC feeding (605 ± 84 vs 278 ± 56 pg/ml, respectively *P*<0.01) and no differences were found in obese mice from either strain (OVAbil vs OVAbilxNLRP3^−/−^) (Figure 3). Despite NLRP3 deficiency this did not affect IL-1β secretion since we found comparable levels of this cytokine in all the experimental groups. We observed a differential secretion of cytokines under different feeding protocol, that is, in the context of a standard chow feeding inflammatory mediators such as IL-6 and CXCL10 were significantly elevated in Ovabil NLRP3-deficient mice as compared to OVAbil NLRP3 sufficient mice (27.8 ± 8 vs 4.6 ± 0.8 and 211 ± 35 vs 94 ± 22, respectively see Figure 3), whereas in the context of obesity a different cytokine profile dominated by IL-13 and TNFα was found (Figure 3). Surprisingly, the levels of type 2 mediators (i.e. IL-4, IL-10 and eotaxin) were negligibly altered (Figure 4) whereas IL-17 was lower in mice lacking NLRP3 regardless of the feeding protocol. Finally, we measured IL-15 given we described a central role in worsening this model in obese mice, here we confirmed our findings; however, the absence of NLRP3 did not result in exacerbated IL-15 (Figure 3, mid panel).

## Discussion

Previous studies reported that biliary inflammation (i.e. PSC) induces expression of NLRP3 in reactive cholangiocytes [12], but the exact role of NLRP3 has poorly been explored *in vivo*. Here, we described that NLRP3 protein seems to be required in order to limit the inflammatory response initiated by the transfer of cholangiocyte-specific T cells. Interestingly, the more prominent defect seemed to be uncontrolled neutrophil expansion and infiltration with simultaneous lower numbers of monocytes. Monocytes and macrophages have been typically recognized as the drivers in liver inflammation [18]; however, findings presented here and our previous report [16] suggest that neutrophils may play an unappreciated and detrimental role in biliary diseases. It has been shown that regulatory cytokines are able to induce NLRP3 expression in renal epithelial cells [19]. Therefore, anti-inflammatory stimuli inducing NLRP3 protein in different subtypes of epithelial cells might enable these cells to display a regulatory function controlling the immunopathology as observed in mice with NLRP3. Bile acids can trigger NLRP3 expression [13,14]; hence, we speculate that modified bile acids resulting upon induction in our model could induce non-canonical activation of NLRP3 although this hypothesis remains to be tested.

The cytokine profile analysis revealed that the severe phenotype displayed in mice lacking NLRP3 resulted from different combinations of inflammatory mediators in SC versus HFD fed mice. In SC fed mice the abscence of NLRP3 lead to an overexpression of IL-6 and CXCL10, both innate inflammatory mediators. It has been shown that one of the effects of NLRP3-mediated IL-1β-secretion is the induction of IL-6 and C-reactive protein [20], thus, a canonical activation pathway of NLRP3 is expected to induce more IL-6, surprisingly, in our hands mice lacking NLRP3 produced comparable low amounts of IL-1β as in the NLRP3-sufficient mice (Figure 3) but enhanced IL-6 suggesting that in this model there is an NLRP3-independent release of IL-6 or an alternate non-canonical NLRP3 activation is ocurring.

On the other hand, we previously reported that obese mice exhibit severe cholangitis related to a combined profile of IL-13/IL-15/IL-17 and indeed IL-15 neutralization attenuated the disease. Intriguingly, NLRP3-deficient obese mice, the group with a devastating liver inflammation, showed no increase of IL-15 and actually suppresed IL-17, but a combined IL-13/TNFα profile was related to this overwhelming disease. This suggests that IL-13 may acquire a highly pathogenic role if combined with other inflammatory cytokines. Intriguingly, there was a selective IL-13 increase amongst type 2 factors suggesting that the cell source of IL-4 and IL-13 may not be shared.

The role of NLRP3 in driving Th-17 responses in the GI tract needs to be clarified since Tian et al*.* reported that activation of NLRP3 promotes IL-17 production in autoimmune cholestasis generated by a dominant negative isoform of the TGFβ receptor II (TGFβRII) [21]. In line with this, Arsenijevic et al*.* recently described a central role of NLRP3 in driving IL-17 in an infectious model of cholangitis as well [22]. In contrast, NLRP3-deficient mice induced with colitis released more IL-17 [9]. Thus, in our immune-mediated model (OVAbil) the absence of NLRP3 caused a IL-17-independent exacerbation of disease.

Thus, deficiency of NLRP3 in OVAbil mice results in an uncontrolled course of the disease upon induction of a cholangiocyte-specific immune response. Further studies are required to elucidate this complex network of immune cells and inflammatory mediators.

## Abbreviations

ALT: alanine aminotransferase
HFD: high-fat diet
OVAbil: transgenic mice expressing an ovalbumin protein fragment in the biliary epithelium
SC: standard chow
TGFβRII: TGFβ receptor II
